# Fundamental Movement Skills among Iranian Primary School Children

**Published:** 2014-12

**Authors:** Bahman Aalizadeh, Hassan Mohamadzadeh, Fatemeh Sadat Hosseini

**Affiliations:** 1 1 Young Researchers and Elite Club, Ardabil Branch, Islamic Azad University, Ardabil, Iran; 2 2 Department of Motor Behavior, Faculty of Physical Education and Sport Science, University of Urmia, Urmia, Iran

**Keywords:** Elementary School Children, Anthropometry, Physical Activity, Socioeconomic status, Manipulative Skills

## Abstract

**Objective:** To determine the relationship between anthropometric indicators, physical activity (PA) and socioeconomic status (SES) with fundamental movement skills (FMS) among Iranian male students.

**Materials and methods:** In this descriptive study, based on SES scores, 241 students (7-10 years) were randomly selected and classified in high, medium and low groups. All children were measured by 8 morphology anthropometric measures. In order to examine a subset of manipulative skills and to measure physical activity and socioeconomic status, Test of Gross Motor Development (TGMD2) and, interviewer-administered questionnaires were used, respectively. The data were analyzed using Pearson correlation and multiple regression.

**Results:** There was a significant positive correlation between SES and body mass index (BMI), while a significant negative correlation existed between PA and BMI. Object control skills were significantly correlated with height, foot length, forearm length, hand length and physical activity.

**Conclusion:** Students with low socioeconomic status were more qualified in movements than other students who were in medium and high socioeconomic status. Therefore, parents need to encourage students to be more active in order to prevent obesity and to facilitate development of object control skills in high socioeconomic status.

## Introduction

Motor skills are the foundation of human behavior and help children to learn new skills in other fields (1). Insufficient motor skill ability in young age affects motor proficiency negatively later in life (2). Therefore, activity can play an important role in childhood growth, socialization, and quality of life (3). Sedentary life style and obesity are products of neighborhood socioeconomic status (4) and they indicate major differences in cultural and socioeconomic aspects which are due to environmental (5), contagion, or stress-related factors in countries like U.S. Other study shows that children of high socioeconomic status have a higher prevalence of obesity compared to students of low socioeconomic status (6). Previous research has shown that socioeconomic status (SES) and language background were positively associated with mastery of some fundamental movement skills (FMS) among school aged children (7). It has been reported that significant negative correlations exist between the locomotor skills subtest and body mass index (BMI) (8). Furthermore, Graf et al. (2004) examined the effect of BMI on the whole body gross motor development in children, and observed the association of overweight or obesity with a poor gross motor development and endurance performance, while children with normal BMI showed better results (9). It is difficult for obese children to move their larger body mass against gravity (10). In addition, overweight children are more likely to have orthopedic complications, such as slipped capital femoral epiphyses (11) and flat feet (10), which may lead to greater pain when performing physical activity and reduced participation. Okley et al. (2001) showed that children with developed motor skills are more physically active and perform better as compared to children with less developed motor skills (12). 

The interactions between these factors— anthropometric indicators, physical activity level, fundamental movement skills and socioeconomic status—are also less well understood. The aim of the present study was to evaluate the association between anthropometric indicators, physical activity level and object control of fundamental movement skills with different socioeconomic status among 7-10 year-old male students living in the city of Urmia, Iran.

## Materials and methods

In this descriptive study, 241 children (age 8.53 ± 1.11) were randomly selected based on socioeconomic status (SES) scores and classified in high (78 student), medium (81 student) and low (82 student) groups. All children and their parents were thoroughly informed about the purposes and contents of the study, and a signed informed consent was obtained from each parent. Furthermore, protocol of the study was approved by the Ethical Committee of the Faculty of Sport and Physical Education, University of Urmia, Urimia, Iran, according to the revised Declaration of Helsinki.

Eight anthropometric characteristics, such as height, weight, BMI, waist hip ratio (WHR), thigh length, foot length, forearm length and hand length were measuredon each subject using the techniques provided by Lohmann et al. (1988) and were prepared in triplicate with the median value used as the criterion (13). Participant's height was recorded via stadiometer (Holtain Ltd., Crymych, Dyfed, UK) to the nearest 0.1 cm while they would stand on the stadiometer with erect posture and bare foot. The horizontal bar of the stadiometer was placed on the participants head and the readings were recorded. To measure the weight the digital standing scales (Model DS-410, Seiko, Tokyo, Japan) to the nearest 0.1 kg was used while participants would stand on the digital weighing machine with minimum cloths and bare foot and records were noted based on the scales of the machine. BMI was then calculated using the following formula: weight (kg)/ [height (m)]^2^. Waist circumference was divided by the hip to determine the waist to hip ratio (WHR). Thigh length was measured from the trochanterion to the tibiale laterale in the right leg while the participants would stand on the box with a little distance between legs. Foot length measurement was performed considering akropodion and pternion points with a sliding caliper in standing position. For measuring elbow length, the distance from the radiale to the stylion was assessed with a tape which was stretched along the radius of flexed elbow. Hand length measurement also was performed considering mid-point below radial and ulnar tuberosity to tip of middle finger while the right hand and fingers were in supination and extension position respectively.

Test of Gross Motor Development-2 (TGMD-2) (Ulrich DA. TGMD-2, 2000) was used to examine a subset of manipulative skills (striking a stationary ball, catch, kick, overhand throw, dribbling and underhand roll) which are related to primary school years activities (14). It is a process-orientated test with test–retest reliability in the range of 0.88–0.96 for FMS research among young children. Each skill consists of 3–5 performance criteria which are scored over two test trials. Scores for each participant were calculated by totaling the correctly performed criteria for two trials of each skill.

For performing the test, an interviewer-administered questionnaire was utilized to know frequency of participation in 13 categories of moderate and vigorous recreational sports, exercise, leisure, and occupational activities over the last 7 days. As described elsewhere (15), physical activity (PA) scores were calculated in exercise units based on frequency and intensity of each activity. Reliability and validity of the instrument is comparable to other activity questionnaires (16). 

The Socio-Economic Status Questionnaire (SES-Q) was used to determine the socioeconomic status level. SES-Q requests participants to respond to the SES subscales including three following items: income, education and occupational status of parents. Therefore, the obtained scores were summed to create a total socioeconomic status score. Previous researches have supported the internal consistency and the construct validity of SES-Q scores in Iranian children (17). In this sample, the internal consistency coefficients for socioeconomic status subscale was α = 0.78. 

Descriptive statistics (means and 95% confidence intervals) were performed to describe each group’s morphological data and all obtained measurements of different anthropometric characteristics. Pearson correlation coefficient (r) was analyzed for understanding the overall relationship between the anthropometric indicators, socioeconomic status and physical activity with fundamental movement skills. To evaluate the importance of anthropometric measures, socioeconomic status, and physical activity on fundamental movement skills, multiple regression analysis was applied. All data were tested for normality using Kolmogorov-Smirnov test, while all data were analyzed using the Statistical Package for Social Sciences software (SPSS) version 16.0 (SPCC Inc., Chicago, IL, USA). The value of p< 0.05 was considered statistically significant.

## Results

The mean skill score (SE) and the prevalence (95% CI) of mastery among boys are shown in table 1. Overall, participants with low SES had a higher object control score than other participants (p < 0.005; 95% CI). Correlation of variables are presented (ą < 0.05) in table 2. Socioeconomic status was significantly correlated with BMI (r = 0.137; p < .033) and weight (r = 0.149; p < 0.020), while significant negative correlations were detected between SES and following anthropometric indicators: thigh length (r = -0.187; p < 0.004), foot length (r = -0.211; p < 0.001), and hand length (r = -0.166; p < 0.010). Physical activity was significantly correlated with BMI (r = -0.190; p < 0.003), weight (r = -0.130; p < .044), foot length (r = 0.178; p < 0.006) and object control skills (r = 0.257; p < 0.000).Object control skills were significantly correlated with height (r = 0.177; p < .006), foot length (r = 0.203; p < 0.002), forearm length (r = 0.249; p < 0.000) and hand length (r = 0.166; p < 0.010).

**Table 1:** Mean (SE) score and confidence intervals (95% CI) of anthropometric indicators, SES, PA and FMS among boys (n = 241)

**Variable**	**Low SES (n = 82)**	**Medium (n = 81)**	**High SES (n = 78)**	
Age(year)	8.54 ± 1.12	8.51 ± 1.13		8.56 ± 1.10
Height(cm)	131.73 ± 8.47	133.32 ± 10.07		132.76 ± 8.36
Weight(kg)	26.39 ± 7.50	29.45 ± 8.62		31.38 ± 6.40
BMI(Kg/m^2^)	17.91 ± 2.63	19.10 ± 2.47		20.64 ± 2.98
WHR	0.84 ± 0.05	0.82 ± 0.07		0.89 ± 0.08
Thigh length (cm)	38.42 ± 3.17	36.58 ± 3.35		33.37 ± 3.28
Foot length (cm)	22.62 ± 1.54	20.76 ± 1.96		20.46 ± 1.81
Forearm length (cm)	20.94 ± 1.88	20.33 ± 1.93		20.59 ± 2.13
Hand length (cm)	14.71 ± 0.99	14.78 ± 1.93		9.25 ± 4.51
Socioeconomic status	8.40 ± 1.07	8.75 ± 0.96		9.58 ± 1.45
Object Control skill	15.83 ± 3.04	14.46 ± 2.68		14.95 ± 2.73
Physical activity	1631.54 ± 749.97	1825.40 ± 1349.79		1500.88 ± 349.78

**Table 2:** Correlations and confidence intervals (95% CI) of anthropometric indicators, SES, PA and FMS among boys (n = 241)

**Variable**	**Object control skill (p, r)**	**Physical activity(p, r)**	**Socio economic status (p, r)**
Height (Kg)	0.006^a^, 0.177	0.997, 0.003	0.381, 0.57
Weight (cm)	0.316, 0.065	0.044^a^, -0.130	0.020^a^, 0.149
BMI (Kg/m^2^)	0.250, -0.074	0.003^a^, -0.190	0.033^a^, 0.137
WHR	0.453, 0.043	0.680, 0.027	0.723, 0.023
Thigh length (cm)	0.305, 0.066	0.174, 0.088	0.004^a^, -0.187
Foot length (cm)	0.002^a^, 0.203	0.006^a^, 0.178	0.001^a^, -0.211
Forearm length (cm)	0.000^a^, 0.249	0.179, -0.151	0.462, -0.048
Hand length (cm)	0.10^a^, 0.166	0.347, 0.061	0.010^a^, -0.166
Socioeconomic status	0.236, 0.077	0.053, -0.125	
Physical activity	0.000^a^, 0.257		

a P < 0.05

Multiple regression analysis was also applied for groups, with anthropometric independent variable (forearm length) as predictors and object control skills as criterion. Indeed, only one (forearm length) of the three predictors was considered as significant determinant for object control skills (F = 20.7; p < .0005; R square = 0.08), while beta coefficient indicated that forearm length had a statistically significant effect on the criterion variables of object control skills (Beta = 282; T = 4.55; p < 0.0005).

## Discussion

The aim of the present study was to examine the relationship between anthropometric indicators, physical activity and socioeconomic status with fundamental movement skills among male students. Participants with high socioeconomic status had higher values in anthropometric indicators, such as BMI and WHR, than students with the middle and low SES. However, we showed increased body segments length and better performance of manipulative skill in participants with low SES, while previous research has shown that SES and language background were positively associated with mastery of some fundamental movement skill(FMS) among school-aged children (7).There was also no significant correlation between SES and object control skills in this study. On the other hand, analysis of Pearson correlation revealed a significant positive association between SES and BMI. In this regard, it has been demonstrated that physical inactivity and obesity are key outcomes related to neighborhood socioeconomic status (4). Other study shows that children with high socioeconomic status have a higher prevalence of obesity compared to students with low socioeconomic status (6).These findings are consistent with those of other studies and suggest that increased economic resources may allow people to purchase and to consume a larger number of high calorie nutrients which then contribute to weight gain. Therefore, the increased consumption of high calorie nutrients may explain some of the positive relationship between SES and BMI. Graf et al. (2004) examined the effect of BMI on the whole body gross motor development in children and observed that overweight or obesity is associated with a poor gross motor development and endurance performance, while children with normal BMI had better results (9).Surprisingly, no significant correlation was found between anthropometric indicators such as BMI and WHR with object control skills. There might be some associations between anthropometric indicators and locomotor skills than object control ones. Since performing locomotor skills needs body mass movement compared to object-control skills, overweight children are not able to execute them well. It is difficult for obese children to move their larger body mass against gravity (10). In addition, overweight children are more likely to have orthopedic complications, such as slipped capital femoral epiphyses (11) and flat feet (10), which may lead to greater pain when performing physical activity and reduced participation. Okley et al. (2001) showed that children who have developed motor skills are more physically active and perform better than children with less developed motor skills (12). This study produced results which corroborate the findings of a great deal of the previous work in this field, while showed a significant positive correlation between physical activity and object control skills. In conclusion**, **students with low socioeconomic status were more qualified in movements as compared with other students belonging to medium and high socioeconomic. Therefore, parents need to encourage students to be more active in order to prevent obesity and to facilitate development of object control skills in high socioeconomic status. 

## Confilict of Interests

Authors declare that they have no conflict of interest.
